# Identification of Lympho-Epithelial Kazal-Type Inhibitor 2 in Human Skin as a Kallikrein-Related Peptidase 5-Specific Protease Inhibitor

**DOI:** 10.1371/journal.pone.0004372

**Published:** 2009-02-03

**Authors:** Ulf Meyer-Hoffert, Zhihong Wu, Jens-Michael Schröder

**Affiliations:** Department of Dermatology, University Hospital Schleswig-Holstein, Campus Kiel, Kiel, Germany; Institut Pasteur, France

## Abstract

Kallikreins-related peptidases (KLKs) are serine proteases and have been implicated in the desquamation process of the skin. Their activity is tightly controlled by epidermal protease inhibitors like the lympho-epithelial Kazal-type inhibitor (LEKTI). Defects of the LEKTI-encoding gene serine protease inhibitor Kazal type (*Spink*)5 lead to the absence of LEKTI and result in the genodermatose Netherton syndrome, which mimics the common skin disease atopic dermatitis. Since many KLKs are expressed in human skin with KLK5 being considered as one of the most important KLKs in skin desquamation, we proposed that more inhibitors are present in human skin. Herein, we purified from human stratum corneum by HPLC techniques a new KLK5-inhibiting peptide encoded by a member of the Spink family, designated as *Spink9* located on chromosome 5p33.1. This peptide is highly homologous to LEKTI and was termed LEKTI-2. Recombinant LEKTI-2 inhibited KLK5 but not KLK7, 14 or other serine proteases tested including trypsin, plasmin and thrombin. *Spink9* mRNA expression was detected in human skin samples and in cultured keratinocytes. LEKTI-2 immune-expression was focally localized at the stratum granulosum and stratum corneum at palmar and plantar sites in close localization to KLK5. At sites of plantar hyperkeratosis, LEKTI-2 expression was increased. We suggest that LEKTI-2 contributes to the regulation of the desquamation process in human skin by specifically inhibiting KLK5.

## Introduction

The skin protects us from water loss and mechanical damage. The surface-exposed epidermis, a self-renewing stratified squamous epithelium composed of several layers of keratinocytes, is most important for the barrier defense against these challenges. Keratinocytes in the outmost stratum corneum (SC) of the epidermis are shed off and replaced by newly differentiated cells originating from epidermal stem cells located in the basal layer. They undergo a specific differentiation process and form the cornified envelope, which is a rigid and insoluble protein and lipid structure with essential properties of the barrier function [1], [2]. Recent discoveries have highlighted the importance of protease-inhibitors and proteases as key players in the desquamation process and in epidermal barrier function.

Human tissue kallikreins, or kallikrein-related peptidases (KLK), are the largest family of trypsin or chymotrypsin-like secreted serine proteases encoded by 15 genes on chromosome region 19q13.4 [3]. At least eight KLKs are expressed in normal skin, among which KLK5, KLK7, KLK8 and KLK14 have been reported to be most important [4]–[6]. KLKs are capable of cleaving corneodesmosomes [7]–[10] and are thought to be key regulators of the desquamation process. Epidermal overexpression of KLK7 resulted in pathologic skin changes with increased epidermal thickness, hyperkeratosis, dermal inflammation, and severe pruritus [11]. The activity of the KLKs is regulated by the pH and specific protease inhibitors in human skin. The importance of epithelial protease inhibitors has been revealed impressively in Netherton Syndrome (NS; OMIM 256500), an autosomal recessive disorder caused by mutations in the serine protease inhibitor Kazal-type 5 (*Spink5*) gene [12]. NS presents as an ichthyosiform dermatosis with variable erythroderma, hair-shaft defects (bamboo hair), atopic features, and growth retardation [13]. Lymphoepithelial Kazal-type-related inhibitor (LEKTI) [14], the product of *Spink5*, includes in its primary structure 15 different serine protease inhibitory domains [14]. The inhibitory functions of LEKTI are highly diverse. Inhibitory activities are directed toward trypsin, plasmin, subtilisin A, cathepsin G, and human neutrophil elastase [15]. Though LEKTI is absent, NS patients can still develop hyperkeratosis – a clinical sign of inhibited desquamation.

Therefore, we speculated that more KLK inhibitors are present in human skin generating a complex network of KLKs and their inhibitors to control the desquamation process. Since KLK5 is thought to be one of the most important enzymes involved in this process, we started a preparative attempt to identify KLK5 inhibitors in human stratum corneum. Herein we report the identification of a new protease inhibitor LEKTI-2 and its gene *Spink9*, which specifically inhibits KLK5.

## Results

### Identification of a new KLK5-inhibiting peptide in human Stratum corneum

**Figure 1:**
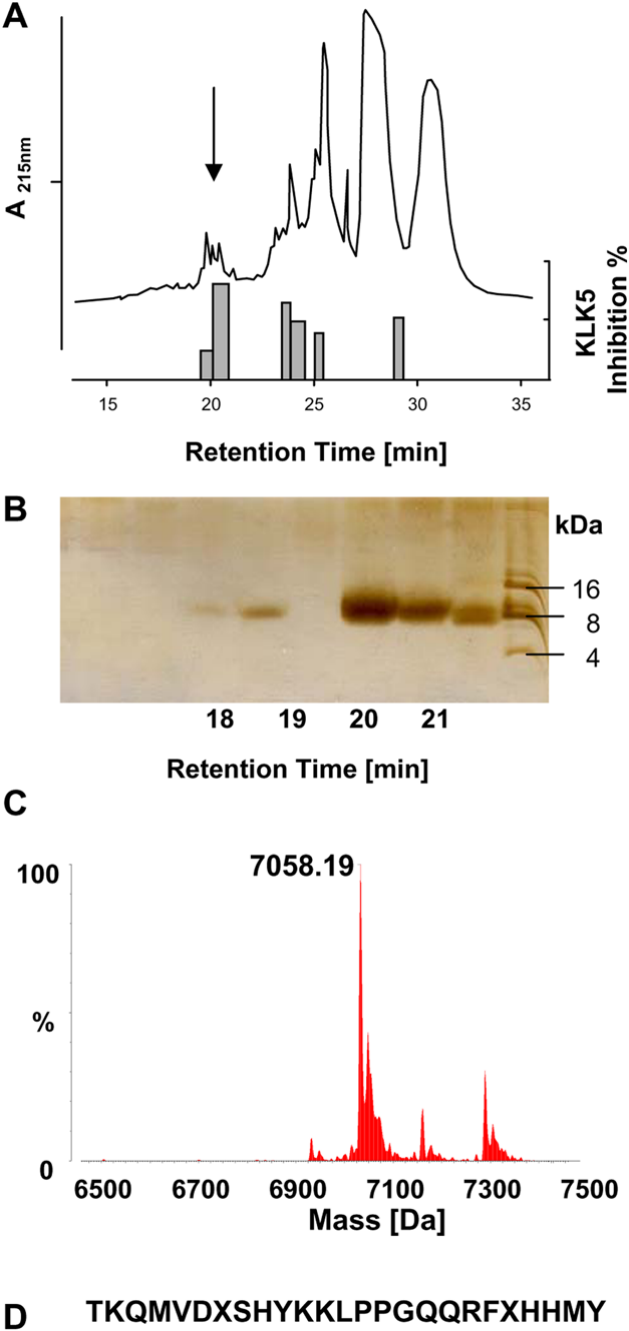
Identification of a new KLK5-inhibiting peptide in human stratum corneum. (A) RP-HPLC separation of extract stratum corneum extracts from human plantar callus. Top, components eluting between 0% and 100% acetonitrile. Bottom, KLK5-inhibiting-activity of the fractions. One out of three similar experiments is shown. Arrow indicates fractions with high activity to absorbance ratio at 20–21 min eluting time. (B) SDS page analyses of fractions eluting between 18 and 22 minutes. Since the fractions were not collected by time but by peaks, distance of fractions is not linear. A dominant peak of around 7 kDa is visible at 20–21 min eluting time. (C) ESI-MS analyses revealed a mass of 7058.18 kDa for the KLK5-inhibiting fraction. (D) Edman degradation resulted in the indicated N-terminal sequence.

To follow the hypothesis that specific inhibitors for KLK5 exist in human skin, extracts from healthy persons' SC were analyzed for KLK5-inhibiting activity. Preparative reverse-phase HPLC (RP-HPLC) was used to separate heparin-bound cationic peptides. Results from KLK5-inhibiting activity revealed a fraction (Fig. 1A), which was further purified by analytical RP-HPLC using a C2C18-column (data not shown). SDS-PAGE analysis (Fig. 1B) of these HPLC fractions, eluting at low acetonitrile concentration and containing KLK5-inhibitory activity showed the presence of a 7-kDa peptide. Electrospray-ionisation mass spectrometry (ESI-MS) analysis (Fig. 1C) resulted in a principal ion corresponding to a mass of 7058.19 Da. Da. N-terminal sequencing of the dominant fraction yielded a sequence of 25 residues (TKQMVDXSHYKKLPPGQQRFXHHMY; Fig. 1D). A blast search using the 25-residue sequence retrieved no matches in any protein/gene/EST databases, suggesting a novel human gene may encode this sequence.

### The KLK5-inhibiting peptide is encoded by *Spink9*

**Figure 2:**
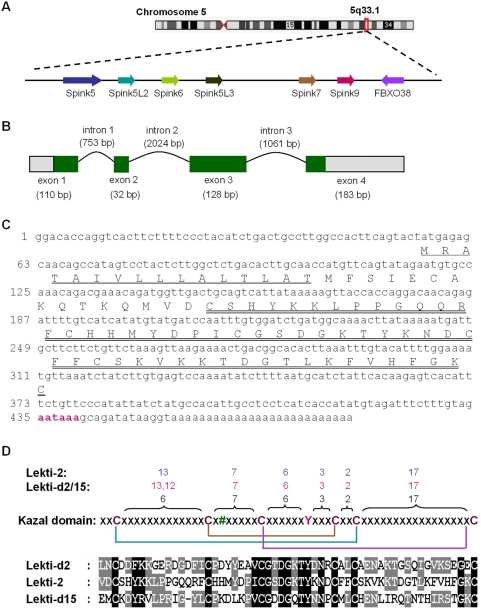
Molecular identification of the *Spink9* gene. (A) Schematic physical map of human SPINK genes locus (5q33.1). Genes are ordered from centromere (left hand side) to telomere (right hand side). (B) Schematic diagram of the *Spink9* gene, based on its cDNA isolated from foreskin-derived keratinocyte identified by RT-PCR. It consists of four exons and three introns. The positions of the exons (boxes) and introns (curve lines) of *Spink9* are deduced by comparing its full-length cDNA sequence with the corresponding genomic DNA. 5′/3′-UTRs and coding sequences are indicated by gray- and green-filled boxes, respectively. (C) The full-length cDNA sequence of *Spink9* and its predicted protein sequence. The N-terminal signal peptide (residues 1–16; underlined) and the Kazal domain (residues 32–86; double-underlined) were detected with the SMART algorithm. The poly(A) signal site was coloured green. (D) Common characteristics of Lekti2 and Lekti. The alignment of the Kazal domains of Lekti-2 and Lekti domains 2 and 15 were generated by using M-COFFEE, displayed by using GeneDoc and shown in the down panel. The middle panel shows a schematic pattern of the typical Kazal domain including conserved tyrosine residue (*Y*) and disulfide bonds [14]. # represents the residue at the P1 site. The residue numbers spacing the cysteine residues are indicated on the top panel for the Kazal domain, LEKTI-2 and the LEKTI domains 2 and 15, respectively.

To identify the gene corresponding to the amino acid sequence, a BLAT search with the N-terminal 25-residue sequence of the novel peptide (where×was replaced by the cysteine residue) against the April 2003 human genome assembly localized this sequence to a chromosome 5 clone RP11-373N22 on 5p33.1 (Fig. 2A). Subsequent analysis of the retrieved RP11-373N22 DNA sequence identified two putative exons exactly encoding the isolated peptide (Fig. 1). Based on the generated theoretical partial DNA sequence, gene-specific primers were designed to perform 3′- and 5′-RACE. The full-length cDNA sequence (453 bp) was completed by combining the overlapping sequences from each PCR product and then confirmed by a long distance PCR (Fig. 2C; GenBank accession No. AY396740). Alignment of the mRNA sequence against human genome sequences clearly indicated that each unique mRNA segment represents an individual exon and that all introns are flanked by the consensus donor and acceptor splice sites conforming to the GT/AG rule (Fig. 2B; data not shown). This gene contains an open reading frame of 261 nucleotides encoding a protein of 86 amino acids; a polyadenylation signal (AAUAAA) is situated 13 nucleotides 5′ of the polyadenine tail (Fig. 2C). By SMART analysis, the 16 residues from the first Met are a leader sequence containing a signal peptide while the last 55 residues correspond to a typical Kazal domain. A BLAST search revealed that this Kazal domain is about 33% identical (50% similar) and 32% identical (40% similar) to domains 2 and 15 of human Lekti, respectively, (Fig. 2D) that is encoded by Spink5, the defective gene in Netherton syndrome [12]. These three Kazal domains possess similar domain patterns, including a conserved tyrosine residue, disulfide bonds and the residue numbers spacing the cysteine residues. Only the P1 residue of the putative active site is different, suggesting they might have different substrate binding modes. Therefore, we designated this novel gene as serine protease inhibitor Kazal-type 9 with the gene symbol Spink9, which was approved later by the HUGO gene nomenclature committee, while its protein product was named lympho-epithelial Kazal-type inhibitor 2 (LEKTI-2).

### *Spink9* is expressed in human skin and in cultured keratinocytes

**Figure 3:**
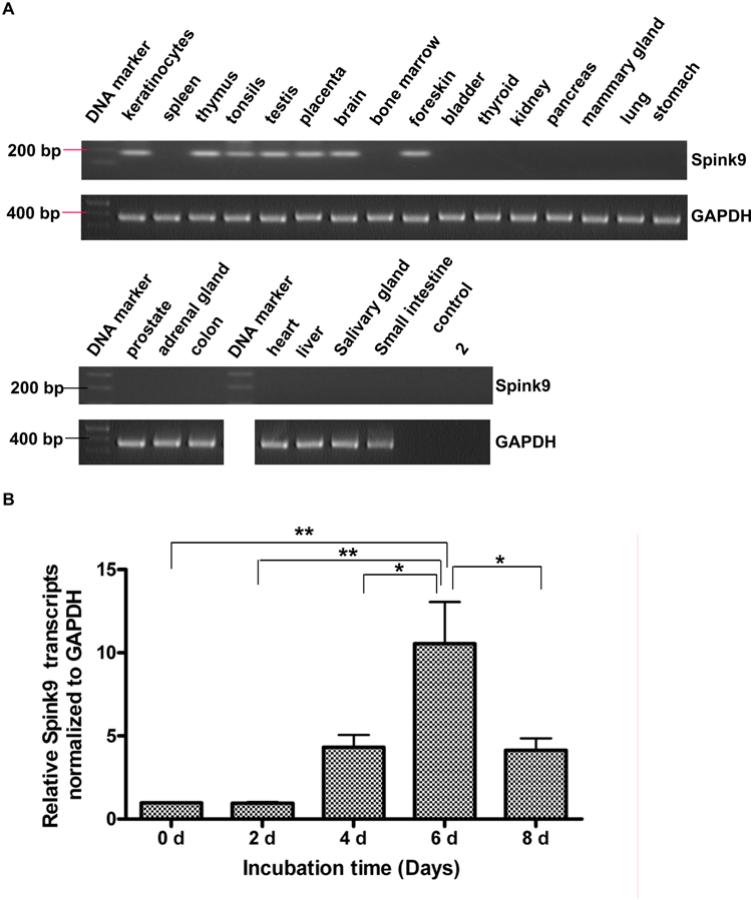
*Spink9* mRNA expression in human skin and keratinocytes. (A) Expression profile of *Spink9* mRNA. Fragments were obtained after RT-PCR amplification on human multiple tissue cDNAs with primers specific to the human *GAPDH* and *Spink9*. Lanes are labelled according to the template tissue. The *Spink9* fragments are of 175 bp in size. H_2_O (no cDNA) and RT-control (no RNA template) were used as negative controls. (B) *Spink9* mRNA expression in cultured primary keratinocytes. Quantitative realtime PCR was conducted on RT-PCR products of total RNA samples collected from keratinocytes treated with 1.0 mM CaCl_2_ for the indicated time. Bar graphs represent the relative mRNA expression of *Spink9* against *GAPDH*. Data are obtained from three independent experiments with different sources of keratinocytes and are indicated as the mean+/−SD. * indicates significant (p<0.05); ** indicates significant (p<0.01).

To investigate the cellular source of LEKTI-2, both RT-PCR and real-time RT-PCR were used to determine its mRNA expression. Expression of *Spink9* mRNA was detected in skin samples from foreskin and cultured primary keratinocytes (Fig. 3A). In addition, its expression was also detected in thymus, tonsils, testis, placenta and brain but not in other tissue samples tested (Fig. 3A). In cultured primary keratinocytes, the expression level of *Spink9* mRNA was increased up to 10-fold over the time course during calcium-induced differentiation, suggesting that *Spink9* is produced by epithelial terminally differentiating keratinocytes.

### LEKTI-2 is expressed at palmar and plantar sites

**Figure 4:**
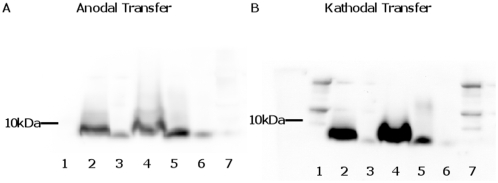
Western blot analyses of LEKTI-2 antibodies. Proteins were separated in a 16.5% SDS-tricine polyacrylamide gel containing 8 M urea and blotted from both, the anode (left panel) and the cathode (right panel) using protein transfer at pH 10.5. Recombinant and natural LEKTI-2 used here have been HPLC-purified, stored below −70°C and used immediately prior to experiments unless otherwise stated. Lanes 1 and 7: Precisions Plus Protein Dual Color standards Biorad (it includes peroxidase-active markers), each 3 µl; lane 2: stratum corneum extract, 20 µl; lane 3: rLEKTI-2 (200 ng); lane 4: stratum corneum extract, 40 µl; lane 5: natural LEKTI-2 from stratum corneum extract (off RP-8-HPLC, see Fig. 1A), 30 µl; lane 6: rLEKTI-2 (200 ng). WB analyses performed with LEKTI-2 antibodies, pretreated for 1 h with 20 µg r LEKTI-2, did not show any LEKTI-2 immunostaining, which further confirms specificity of the LEKTI-2 antibodies. Note that cathodal transfer seems to be more efficient than anodal protein transfer. A representative out of three independent experiments is shown.

**Figure 5:**
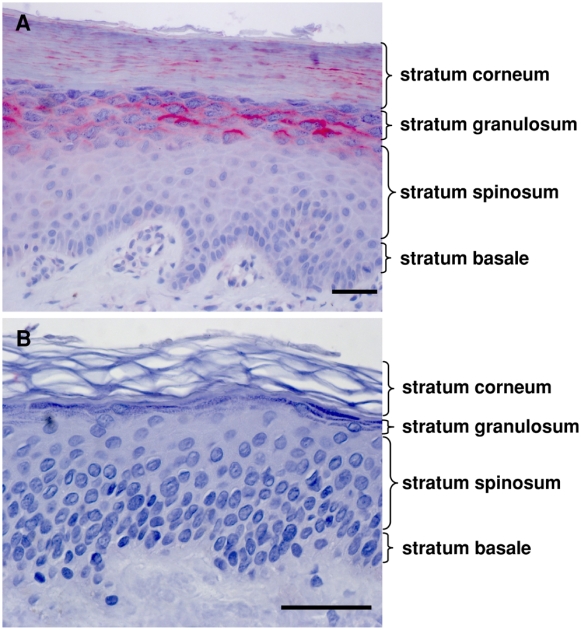
LEKTI-2 is expressed at palmar and plantar sites. Immunohistochemical staining (Vector red) of LEKTI-2 of paraffin-embedded human skin samples using polyclonal antibodies. Only palmar and plantar localizations (A, n = 8) exhibited obvious LEKTI-2 immunoreactivity at the stratum granulosum and stratum corneum. Other localizations (e.g. trunk, B, n = 16) did not reveal LEKTI-2 immunoreactivity. Bars indicate 50 µm.

To analyze LEKTI-2 protein expression, we generated affinity-purified polyclonal LEKTI-2 antibodies. Westernblot analyses performed with rLEKTI-2, purified natural LEKTI-2 and stratum corneum extracts revealed antigen specificity of the antibodies (Fig. 4), which was further confirmed by blocking experiments using recombinant LEKTI-2. Subsequently, LEKTI-2 immunohistochemistry was performed to localize LEKTI-2 expression in human skin samples. LEKTI-2 immunoreactivity was detected in the stratum granulosum and SC of human skin at the palms (inner sides) of hands and feet (n = 8) but no visible immunoreactivity was detected at other sites of healthy human skin (n = 16) (Fig. 5).

### Recombinant LEKTI-2 is a specific KLK5-inhibitor

**Figure 6:**
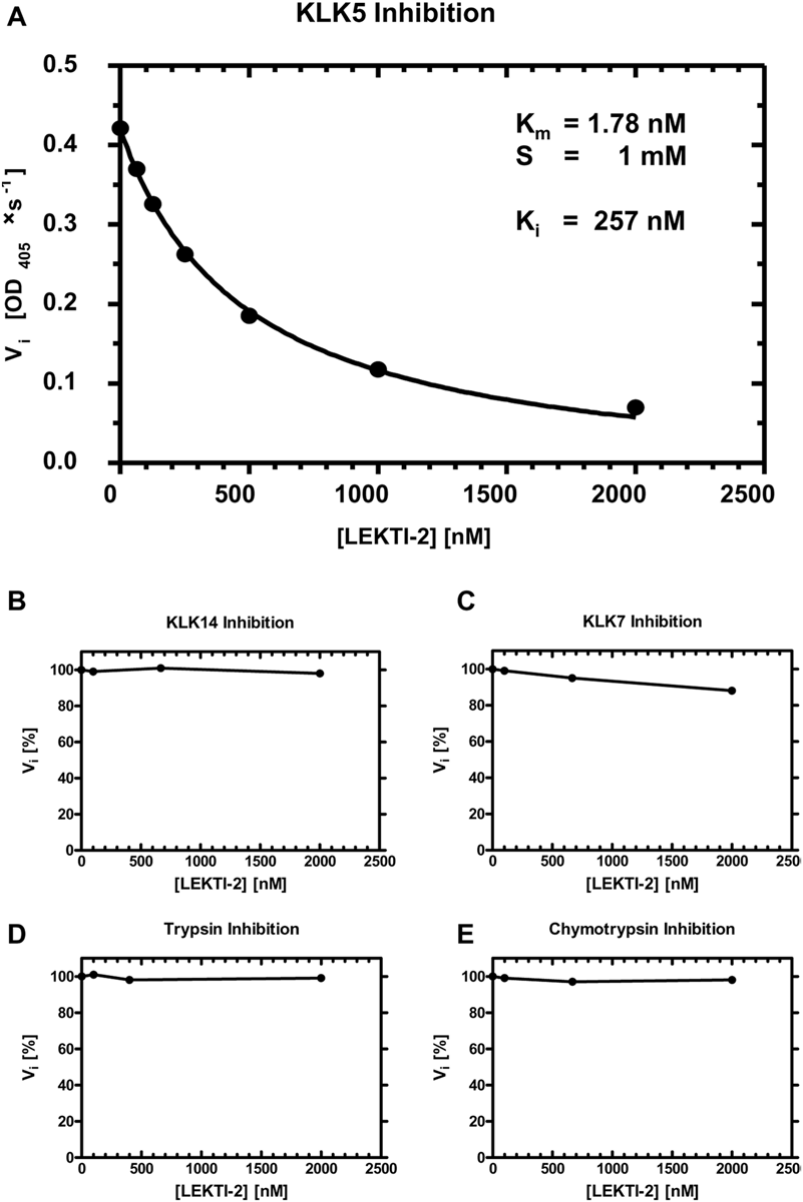
Inhibition of KLK5 by rLEKTI-2. (A) Enzyme kinetic of KLK5 (5 nM) and LEKTI-2 (0 to 2000 nM) in comparison to no inhibition of KLK14 (B), KLK7 (C), trypsin (D) and chymotrypsin (E). The substrate for KLK5 was MeO-Suc-Arg-Pro-Tyr-pNA (1 mM). Points represent mean of triplicate experiments and the solid line represents the fitted curve according to equation of Baici model [33].

To verify the protease inhibition of the purified LEKTI-2, recombinant LEKTI-2 was tested for its protease inhibitory activity. KLK5 was inhibited in a dose dependent manner. Assuming full competitivity of binding (alpha = infinitive) Baici Model was used to calculate K_i_ (approximately 250 nM, Fig. 6). Interestingly, no other tested serine proteases, which include KLK7, KLK14, trypsin and chymotrypsin were inhibited by LEKTI-2 (Table 1, Fig. 6).

**Table 1:** Protease Inhibition by rLEKTI-2.

Proteinase (final concentration)	LEKTI-2 (nM)	Inhibition (%)	Substrate (0.33 mM)
Bovine Trypsin (2 nM)	400	0	N-(p-Tosyl)-Arg-Gly-Val 5-nitroanilide
Cathepsin G (1 nM)	666	0	N-Succinyl-Ala-Ala-Pro-Phe p-nitroanilide
Chymase (2 nM)	666	0	N-Succinyl-Ala-Ala-Pro-Phe p-nitroanilide
Human Chymotrypsin (2 nM)	400	0	3-Carbomethoxypropionyl-Arg-Pro-Tyr p-nitroaniline
KLK14 (2 nM)	400	0	N(p-Tosyl)-Arg-Gly-Val 5-nitroanilide
KLK5 (5.3 nM)	400	91.8	N(p-Tosyl)-Arg-Gly-Val 5-nitroanilide
KLK7 (15.8 nM)	400	0	3-Carbomethoxypropionyl—Arg-Pro-Tyr p-nitroaniline
Human leukocyte elastase (2 nM)	400	0	N-Methoxysuccinyl-Ala-Ala-Pro-Val p-nitroanilide
Human Plasmin (2 nM)	400	0	N-(p-Tosyl)-Gly-Pro-Lys 4-nitroanilide
Human Thrombin (1 nM)	400	0	N-(p-Tosyl)-Gly-Pro-Arg p-nitroanilide
Matriptase (0.5 nM)	400	0	H-D-Ile-Pro-Arg p-nitroaniline

### LEKTI-2 and KLK5 are in close localization in vivo

**Figure 7:**
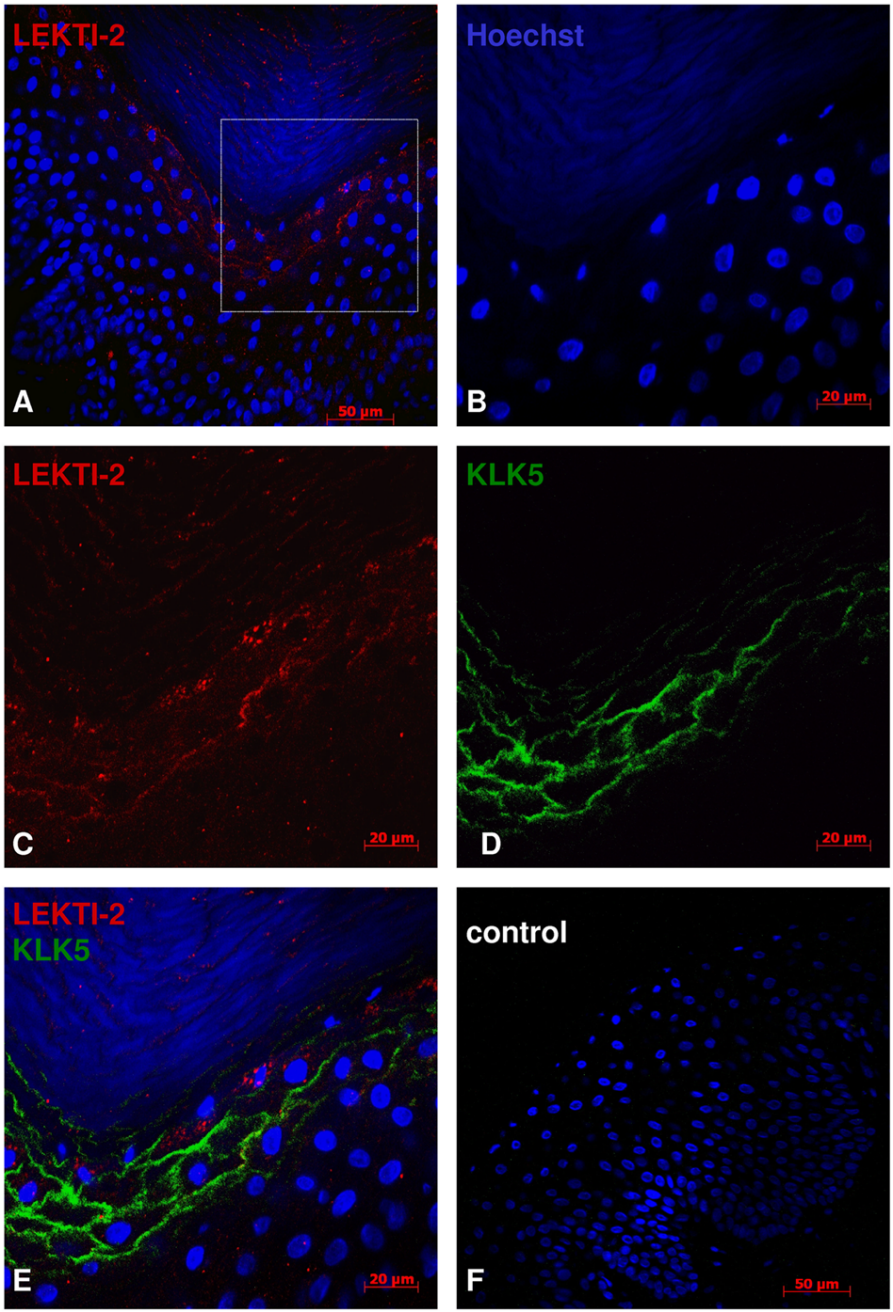
LETKI-2 and KLK5 are expressed at the stratum granulosum of palmar skin. Immunofluorescence localization of LEKTI-2 (red) and KLK5 (green) in human skin. Nuclei staining was done using Hoechst 33258 reagent. (B–E) shows the magnification of the white-square area of (A). LEKTI-2 staining showed granular structures inside the keratinocytes at the stratum granulosum (C) with a faint intercellular staining pattern and remaining immunoreactivity inside the stratum corneum. KLK5 staining (D) exhibited only intercellular staining at the stratum granulosum. Comparative localization of LEKTI-2 and KLK5 is shown in the merged image (D). (E) shows the control omitting the first antibody.

To visualize whether LEKTI-2 and KLK5 localize to the same site in human skin, fluorescent microscopic analyses were performed. LEKTI-2 fluorescent staining revealed granular-like structures inside the keratinocytes at the stratum granulosum and an intercellular staining in this area and in the SC (Fig. 7). KLK5-staining revealed intercellular expression pattern at the same area. However, a clear co-localization was not observed.

### LEKTI-2 is highly expressed at sites of hyperkeratosis

**Figure 8:**
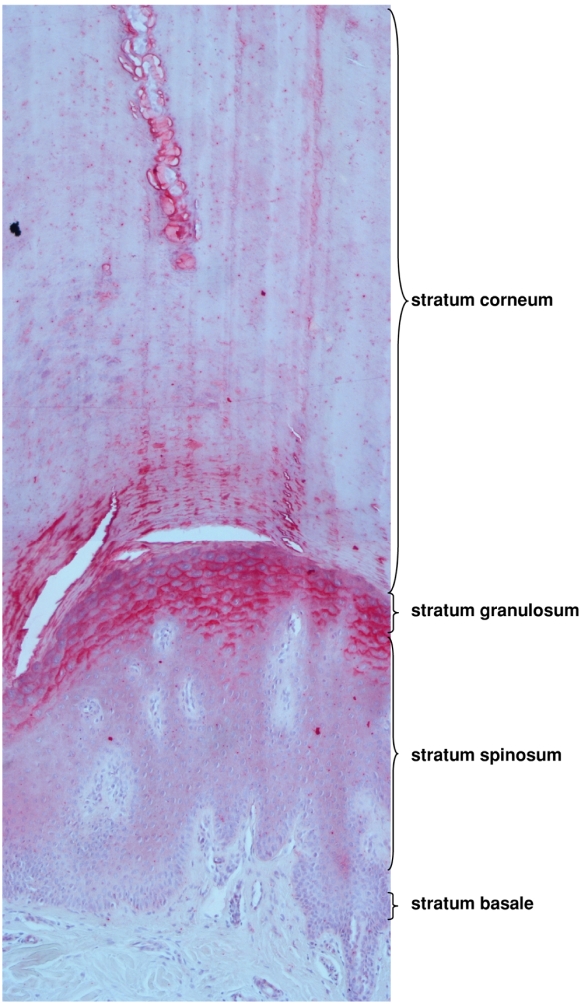
LEKTI-2 is highly expressed at sites of hyperkeratosis. Immunohistochemical staining (Vector red) of LEKTI-2 of a paraffin-embedded section of a clavus. Notice the enormously enlarged stratum corneum (hyperkeratosis) in the upper two third of the picture with a sweat gland sectioned at the upper first third. Clefts between the stratum corneum and the stratum granulosum are artefacts derived from histology processing. The upper part of the living epidermis, the stratum granulosum, exhibits a massive increase in LEKTI-2 immunoreactivity (red). A representative result out of three different samples of clavi is shown.

Hyperkeratosis at the palmar sites can occur due to increased local mechanical pressure and lead to hyperkeratosis in the form of clavus. Interestingly, LEKTI-2 immunoreactivity was shown to be markedly induced at lesions of clavi (Fig. 8). Since KLK5 is one of the major proteases for desquamation, increased LEKTI-2 expression at the sites of clavi might contribute to the hyperkeratosis of these lesions.

## Discussion

In this study we aimed to identify major substances that might contribute to the epithelial barrier shield by inhibiting the epidermal serine protease KLK5. We identified a new peptide termed LEKTI-2 as a specific inhibitor for KLK5, which is encoded by *Spink9*, a novel member of the Spink gene family. Our findings give evidence for the importance of LEKTI-2 in epidermal desquamation and provide new insight to the complex protease-protease inhibitor interaction in human skin.

LEKTI-2 expression shows some similarities to the expression of LEKTI, which was demonstrated to be expressed in lamellar bodies, likely the granular-like structures in our fluorescent staining, and secreted into the intercellular space, in the uppermost stratum granulosum [16]–[18]. Electron microscopy studies revealed that LEKTI and KLK7 are transported separately in the lamellar granule system and are co-localized in the extracellular spaces [18]. Our findings of LEKTI-2 and KLK5 expression are accordable to those results but need further evaluation by electron microscopy. However, LEKTI-2 expression was only detected in our studies at palmar and plantar sites where a rigid SC is needed to protect the hands and feet from mechanical damage. The fact that we did not find LEKTI-2 immunoreactivity at other sites, though low mRNA expression was detectable in skin samples, points to a minor role of LEKTI-2 in non-plantar skin compared to LEKTI, which is expressed throughout the entire skin. The circumstance that we used plantar human callus as the natural source of KLK5 inhibitors was therefore beneficial for the identification LEKTI-2. The enhanced expression of LEKTI-2 in plantar clavus corroborates the hypothesis that LEKTI-2-mediated KLK5 inhibition results in suppressed desquamation. Clavi are often induced by abnormal local mechanical pressure due to malformation of feet bones or tight footgear. It will be interesting to study how LEKTI-2 expression is induced by these mechanical forces. Mechanical stress represents an important part of signaling in skin. Indeed, *in vitro* it induces phosphorylation of keratin K6 and EGFR [19] and clustering of beta1-integrins [20], and activates ERK1/2 [19] as well as Akt, one of the kinases known to suppress apoptosis [21].

Most notably, LEKTI-2 exhibited only inhibiting activity against tryptic KLK5 but not against the chymotryptic KLK7, tryptic KLK14 or all other serine proteases tested including trypsin and chymotrypsin. LEKTI-2 activity differs in this respect from that of LEKTI, which contains multiple Kazal domains exhibiting highly diverse inhibitory functions beyond others against trypsin, plasmin, subtilisin A, cathepsin G, and human neutrophil elastase [15]. Therefore, the functions of both Kazal-type inhibitors are suspected to be different. Overall trypsin-like and/or chymotrypsin-like activities resulting mainly from KLKs are considerably elevated in the skin of *Spink5*-deficient mice [22] and in NS patients [4], [23]. The elevated activities cause increased degradation of corneodesmosomal cadherins in NS patients [24]. It was shown that KLKs are capable of cleaving corneodesmosomes, [7]–[10]. Furthermore, Kallikrein-mediated proteolysis regulates the antimicrobial effects of cathelicidins in skin [25] and contributes to the pathogenesis of rosacea [26]. Moreover, KLK5 and KLK14 haven been reported to activate the protease activated receptor (PAR)-2 [27], a signaling receptor in epidermal inflammation [28] and regulator of epidermal barrier function [29]. Altogether, these accumulating data strongly suggest that in skin, KLKs are desquamation-related serine proteases and that the balance between serine proteases and inhibitors may be essential, not only for steady desquamation but also for skin barrier function and inflammation. Regulation of KLKs by endogenous proteinase inhibitors like LEKTI and LEKTI-2 might therefore have therapeutic potential in inflammatory skin diseases.

The K_i_ of LEKTI-2 against KLK5 observed herein can be considered as preliminary since more in depth analysis of inhibition was not possible in this study due to limited access to KLK5. Several LEKTI domains have been reported to inhibit KLK5 in various reports. In comparison, the determined K_i_ of LEKTI domains to inhibit KLK5 was in the range of 3 nM (domain 8–11) to 120 nM (domain 9–15) [9], [30], [31]. It will be very interesting to study the inhibition of other KLKs by LEKTI-2 in future investigations. KLKs are tryptic and chymotryptic enzymes though a detailed comparison of their activities, substrates and specific inhibitors has not been done systematically. As the most intensively studied KLK, KLK3 (also known as prostate-specific antigen), is a chymotryptic enzyme, similar in this respect to KLK7, which was not inhibited by LEKTI-2. Since *Spink9* mRNA was detected in other organs like tonsils, testis, placenta and brain, but not in the prostate, it remains speculative what function LEKTI-2 might have in other organs. The only LEKTI-2 target enzyme identified so far is KLK5, which was reported to be expressed at high levels (100–1000 ng/g tissue) in breast, testis, salivary glands and thyroids but in the skin at highest reported levels (above 10,000 ng/g tissue) [32]. Detailed investigations of LEKTI-2 function in other organs are required to clarify its role outside the skin.

In summary, we identified a new Kazal-type inhibitor LEKTI-2 and its gene *Spink9* and showed its expression in human skin. LEKTI-2 exhibited specific inhibition against KLK5, which might be important in desquamation especially at palmar and plantar sites.

## Materials and Methods

### Material

All experiments were performed according to the Declaration of Helsinki protocols and under protocols approved by the ethics committees of the Medical Faculty of the Christians Albrechts University Kiel, (Schwanenweg 20, D-24105 Kiel). Normal skin specimens were taken from routine clinical work at the Department of Dermatology, UKSH Kiel, and represent tumor-free margins of benign melanocytic tumors surgically removed from patients. Specimens of clavi were removed therapeutically before written informed consents were received from the patients. Restriction endonucleases were from New England Biolabs (Frankfurt, Germany). KLK5, 7, and 14 were purchased from R&D Systems, Minneapolis, MN. All other proteases, primers, substrates and chemicals were purchased from Sigma–Aldrich (Taufkirchen, Germany), if not otherwise indicated.

### Protease Inhibition Assays

All protease assays were performed measuring chromogenic substrate release by proteases. Following buffers were used: KLK5: 50 mM Tris-HCl, pH 8.0, at 21°C; KLK7, KLK14:50 mM Tris-HCl, 0.15 M NaCl, pH 8.0, at 21°C. All other proteases (Table 1) were measured in the buffer recommended by the manufacturer. Specific concentrations of protease, substrate and inhibitor are indicated in Table 1. The changes of absorbance at 405 nm were followed up to 16 h in comparison with enzyme-free controls. Inhibition of proteases was measured after preincubating the enzyme with inhibitor or HPLC fraction for 15 min at 21°C. K_m_ was determined for KLK5 using various concentrations of substrate. K_i_ was calculated using Baici Model [33] assuming full competitivity of binding.

### Isolation of KLK5 inhibitor from human stratum corneum

Total proteins were extracted from heel stratum corneum, a clinical circumstance when desquamation might be inhibited by KLK-inhibitors, with an acidic buffer. Briefly, pooled heel stratum corneum (80–120 grams) was extracted with acidic ethanolic citrate buffer as described [34]. After diafiltration (Amicon filters, cut off: 3 kDa) against 10 mM Tris/citrate buffer, pH 8.0, extracts were applied to a heparin-sepharose cartridge (10×5 mm, Pharmacia, Freiburg, FRG), previously equilibrated with the diafiltration buffer. After washing, bound proteins were eluted with 2 ml 2 M NaCl in 0.1 M Tris/citrate buffer and the heparin-bound material was further diafiltrated against 0.1% (v/v) trifluoroacetic acid (TFA) in HPLC grade water. Heparin-bound material was purified by preparative wide-pore reversed phase high-performance liquid chromatography (RP-HPLC) using a column (300×7 mm, C8 Nucleosil, 250×12.6 mm, Macherey and Nagel, Düren, Germany) that was previously equilibrated with 0.1% (v/v) TFA in HPLC grade water containing 20% (v/v) acetonitrile. Proteins were eluted with a gradient of increasing concentrations of acetonitrile containing 0.1% (v/v) TFA (flow rate: 2 ml/min). Aliquots (10–30 µl) of each fraction were lyophilized, dissolved in 5 µl 0.1% (v/v) aqueous acetic acid and tested for protease-inhibiting activity.

Fractions containing KLK5-inhibiting activity, eluting at low (25%) acetonitrile were further purified by micro-C2/C18-RP-HPLC and tested for KLK5-inhibiting activity. Protease-inhibiting activity-containing HPLC fractions were further analyzed by Electrospray-ionization mass spectrometry (ESI-MS) in the positive ionization mode with a quadrupole orthogonal accelerating time-of-flight mass spectrometer (QTOF-II hybrid mass spectrometer; Micromass, Manchester, United Kingdom). Concentrations of proteins present in HPLC fractions were estimated via UV-absorbance integration at 215 nm using ubiquitine for calibration. The N-terminal amino acid sequence of the principal protein was determined using a pulsed-liquid-phase 776 automated protein sequencer (Perkin Elmer Applied Biosystems, Massachusetts, USA).

### *In silico* analyses

<1?twb=.3w?>Homology search was done using the BLAT algorithm [35] as provided by the UCSC Genome Browser (http://genome.ucsc.edu/cgi-bin/hgBlat) and the BLAST algorithm [36] as provided by the Ensembl server (http://www.ensembl.org/Multi/blastview). Subsequent sequence manipulations utilized the online BLAST 2 Sequences [37] (http://www.ncbi.nlm.nih.gov/ blast/bl2seq/bl2.html). Splice site analysis was performed using the FSPLICE program implemented at the SoftBerry server (http://www.softberry.com/berry.phtml). Protein domains were discovered on the SMART server [38]. Multiple sequence alignments were performed using the M-coffee program [39] and edited with GeneDoc (http://www.psc.edu/biomed/genedoc).

### Rapid amplification of cDNA ends (RACE)

Total RNA was obtained from cultured human foreskin-derived keratinocytes using TRIzol reagent (Invitrogen, Hamburg, Germany). After treatment with RNase-free DNase I (Roche Diagnostics, Mannheim, Germany) to exclude contamination with genomic DNA, 3 µg of DNA-free total RNA was used for the first-strand cDNA synthesis for RACE using SMART RACE cDNA Amplification Kit (BD Bioscience Clontech, Heidelberg, Germany) according to the manufacturer's protocol. To obtain the 5′-end of *Spink9* cDNA, a 5′-RACE was performed with a gene-specific antisense primer (5′-TGC CAT CAG ATC CAC AAA TTG GAT CAT AC-3′) and a universal primer mix (10× UPM) essentially according to the manufacturer's protocol. 5′-RACE PCR reaction cycles were performed with an annealing temperature of 68°C and 35 cycles. To obtain the 3′-end of *Spink9* cDNA, the first round PCR was performed with a gene-specific sense primer (5′-GCC AAA CAG ACG AAA CAG ATG GTT GAC T-3′) and 10× UPM. Subsequently, 0.5 µl of PCR products was used as a template for a nested PCR with a nested gene-specific sense primer (5′-ACC ACC AGG ACA ACA GAG ATT TTG TCA TC-3′) and a nested universal primer (NUP) under the following conditions: 1 min at 95°C, 30 cycles of 20 s at 95°C and 3 min at 70°C, and a final extension of 10 min at 70°C. The amplified fragment was gel purified and subcloned into the pGEM-T vector (Promega, Mannhein, Germany) followed by fully sequencing in both directions.

### mRNA expression analyses

A total of 2 µg of total RNA from human skin samples or cultured foreskin-derived keratinocytes was reverse transcribed with an oligo(dT)_18_ primer and Superscript II RNaseH^−^ reverse transcriptase (Invitrogen, Hamburg, Germany). For following PCR analysis, one pair of gene-specific PCR primers (forward primer: 5′-GAC ACC AGG TCA CTT CTT TTC CCT ACA TC-3′; reverse primer: 5′-TCT ACA TAT GGT GAT GAG TAG GCA ATG TG-3′) were designed to amplify a 422-bp product and to span all three exon-intron boundaries. PCR amplifications were carried out for 35 cycles of 20 s at 95°C followed by 30 s at 72°C using Advantage 2 Polymerase Mix (BD Bioscience Clontech, Heidelberg, Germany). PCR products were analyzed by 2.0% agarose gel electrophoresis. Because of low expression of *Spink9* mRNA in most tissues and cells, all PCR products above were then diluted 20-fold in water and used for nested PCR and real-time PCR. For nested PCR, another pair of intron-spanning primers (forward primer: 5′-ACT TGC AAC CAT GTT CAG TAT AGA-3′; reverse primer: 5′-AAC TTT AGA ACA GAA GAA GCA ATC A-3′) was designed to amplify a 175-bp product. PCR products were then analyzed by 2.0% agarose gel electrophoresis.

As an internal control of cDNA templates, the housekeeping gene *GAPDH* (glyceraldehyde phosphodehydrogenase) was assessed with each cDNA in a separate PCR reaction. For quantitative real-time RT-PCR, assay was carried out with the first primer pair as above and the SYBR® Premix Ex Taq™ Kit (Takara Bio, Heidelberg, Germany) in a fluorescence thermocycler following the instructions of the manufacturer (LightCycler, Roche Molecular Biochemicals, Hamburg, Germany). During the evaluation phase of the assay, amplicons were analyzed by 2.0% agarose gel electrophoresis and, where necessary purified and sequenced to confirm their identity. For calculation of the relative transcripts amplification, the housekeeping gene *GAPDH* was performed with each cDNA in a separate PCR reaction. The data from triplicate samples were analyzed with software (GraphPad Prism 4) and expressed as mean±SD of mRNA in question relative to that of GAPDH. The statistical analyses were performed with One-way ANOVA method and p<0.05 was considered significant while p<0.01 was considered very significant.

### Recombinant protein production

The recombinant expression of *Spink9* cDNA in *E. coli* was performed by molecular subcloning of *Spink9* cDNA into the prokaryotic expression vectors pET-32a (Novagen, North Ryde, Australia). The purified form of LEKTI2 (amino acid 26 to 86) was generated as PCR fragments (Primer sequences are available upon request) by using *Pfu* DNA polymerase (Promega, Mannhein, Germany). PCR products were double-digested with *Bgl*II and *Not*I prior to be cloned into the similarly double-digested pET-32a vectors. Clones were sequenced to check for any mutation that might have been misincorporated during the amplification. The expression construct was transformed into *Escherichia coli* (*E. coli*) BL21*trxB*(DE3)*pLysS* cells (Novagen) and selected on Luria-Bertani (LB) agar plates containing carbenicillin (100 µg/ml), chloramphenicol (34 µg/ml) and kanamycin (15 µg/ml). Protein expression was induced with 1 mM IPTG (isopropyl thio-β-D-galactoside) for a 3 h. After incubation, cells were harvested by centrifugation and resuspended in 1×LEW buffer (50 mM NaH_2_PO_4_, 300 mM NaCl, pH 8.0). Resuspended cells were subjected to one cycle of freeze-thawing and sonicated on ice until complete lyses. After centrifugation at 15,500×g for 30 min, the clarified supernatant was applied to Protino®Ni prepared columns (Macherey-Nagel, Dueren, Germany) and the polyhistidine-tagged protein was eluted with 1×elution buffer (50 mM NaH_2_PO_4_, 300 mM NaCl, 250 mM imidazole, pH 8.0). The further purification of the fusion protein was achieved by reversed phase high-performance liquid chromatography (RP-HPLC) using preparative wide-pore C8 RP-HPLC with a column (SP250/10 Nucleosil 300-7 C8; Macherey-Nagel) that was previously equilibrated with 0.1% (v/v) TFA in HPLC-grade water containing 10% acetonitrile. Proteins were eluted with a gradient of increasing concentrations of acetonitrile containing 0.1% (v/v) TFA (flow rate, 3 ml/min). Fractions containing UV (215 nm)-absorbing material were collected, lyophilized and analyzed by ESI-QTOF-mass spectrometry (Micromass, Manchester, U.K.). The His-tagged fusion protein purified from HPLC-RP8 was cleaved from its His-tag with SUMO protease according to the manufacturer's suggestion (Lifesensors Inc., Pennsylvania, USA). The digestion mixture contained 1 unit of SUMO protease per 100 µg of the fusion protein in a volume of 500 µl of 1×PBS buffer and was incubated for 2 h at 30°C. The dialyzed sample was adjusted to a pH value of 3.0 to 4.0 and then purified by centrifugation. The supernatant was collected and injected onto a Jupiter-5µ-C4-300A HPLC column (Phenomenex, Aschaffenburg, Germany) equilibrated with 0.1% TFA in water. Peptides were eluted with a gradient of increasing concentrations of acetonitrile containing 0.1% (v/v) TFA (flow rate, 0.5 ml/min). Fractions of each peak were collected, lyophilized and analyzed by ESI-QTOF-mass spectrometry.

### Production of antibodies

Polyclonal antiserum was generated in a goat against a full length peptide with the amino-acid sequence of human LEKTI-2 (amino acid 26 to 86). A total of 1.0 mg of protein mixture including 500 µg of fusion protein (pET-32a-LEKTI-2-3) and 500 µg of HPLC-purified recombinant peptide was conjugated by the glutaraldehyde method to maleimide-activated keyhole limpet hemocyanin (KLH) (protein-KLH 1 ∶ 1, w/w) and subsequently mixed with 500 µg of pET-32a-lekri-2-3 for use as immunogens. Immunization of a goat was carried out four times on days 0, 14, 28 and 35. Goats were bled 2 weeks after the last booster. The serum was separated from the clot and stored at −70°C until required. Antisera were affinity-purified by absorption against rLEKTI-2-3 that was covalently bound to HiTrap NHS-activated HP 1 ml columns (Amersham Biosciences, Freiburg, Germany). Specificity was tested by immuno-dot analyses and Western blot analyses using purified rLEKTI-2, purified natural LEKTI-2 and stratum corneum extracts.

### Western blot analyses

For Western-blot analysis healthy persons' stratum corneum extract, HPLC-fractions containing natural LEKTI-2 or recombinant LEKTI-2, were loaded onto a 16.5% SDS-tricine polyacrylamide gel containing 8 M urea. Proteins were transferred to a Protran-nitrocellulose membrane (Schleicher & Schuell BioScience, Dassel, Germany), blocked for 1 h in blocking buffer (5% (w/v) nonfat powdered milk in PBS+0.05% Tween), then incubated for 18 h at 4°C in 3% (w/v) nonfat powdered milk in PBS+0.05% Tween containing 1∶1,250 affinity-purified polyclonal LEKTI-2 antibody. The membrane was washed with PBS+0.05% Tween six times for 5 min each, then incubated for 1 h in 3% (w/v) nonfat powdered milk in PBS+0.05% Tween containing 1∶20,000 dilution of goat anti-mouse IgG HRP conjugate (Dianova, Hamburg, Germany). After six times washing steps as before the membrane was incubated for 5 min with chemiluminescent peroxidase substrate (Sigma, Taufkirchen, Germany) and visualized using a Diana III cooled CCD-camera imaging system (Raytest, Straubenhardt, Germany). Densitometric quantifications were performed using AIDA evaluation software (Raytest).

### Cell culture

Foreskin-derived primary keratinocytes were prepared from neonatal foreskin after surgery following established methods [40] and were cultured in Epilife medium in 75-cm^2^ flasks (BD Biosciences, Heidelberg, Germany) in a humidified atmosphere with 5% CO_2_. For stimulation and RNA isolation, cells were grown in 12-well tissue culture plates (BD Biosciences) and were used after the second passage at a confluence of 70–80%. Stimulation was performed for the indicated time with 1.0 mM of freshly prepared CaCl_2_ for the indicated time.

### Immunohistochemistry

Fixation of the tissue samples was performed in 4% paraformaldehyde. Paraffin sections (5 µm) of the tissue samples were deparaffinised and rehydrated before heat-induced antigen retrieval was performed in 0.01 M citrate buffer (pH 6.0). The slides were blocked with normal rabbit serum (1∶75, Dako Cytomation, Glostrup, Denmark) before staining. Immunohistochemical staining was performed at room temperature for one hour using with affinity-purified polyclonal goat anti-lekti-2 antibody (1∶200 dilutions. A biotinylated secondary rabbit anti-goat IgG (1∶100, Dako Cytomation) antibody was used, followed by incubation with Vector Universal ABC Alkaline Phophatase Substrate Kit (Vector, Burlingame, CA, USA) developed with Vector NovaRED Substrate (Vector) and counterstained with hematoxylin. Specificity test of the anti- lekti-2 antibody was performed by using recombinant lekti-2 peptides to block the primary antibody. Negative controls were established by using preimmune goat sera to stain sections.

### Immunofluorescence

To show the potential spatial relationships between LEKTI-2 and KLK5, the paraffin-embedded skin sections (5 µm) were blocked with 10% normal goat and rabbit sera (Vector) in TBS containing 0.1% BSA and 0.2% glycine after standard rehydration. Sections were incubated with a mixture of anti-LEKTI2 antibody (1∶100 dilution) and a rabbit anti-KLK5 antibody (1∶200 dilution; Jackson R&D systems, Minneapolis, MN). After washing, they were incubated with a mixture of secondary antibodies (Cy3-coupled pig-antigoat IgG and Cy2-coupled chicken-antirabbit IgG, diluted 1∶400 each; Dianova, Hamburg, Germany) for 1 h at room temperature. Sections were counterstained with the DNA-selective bisbenzimide dye (blue; Hoechst 33258). To exclude artificial autofluorescence secondary to the preparation of the sections, control sections were stained without primary antibodies and no unspecific labeling was observed following incubation with secondary antibodies (data not shown). Slides were analyzed using a confocal laser scanning microscopy (Zeiss, LSM 510 UV, Jena, Germany).
